# Laparoscopic Surgery for Sigmoid Colon Cancer in a Patient With a History of Two Renal Transplantations and Peritoneal Dialysis

**DOI:** 10.7759/cureus.56209

**Published:** 2024-03-15

**Authors:** Daisuke Tomita, Kodai Nagakari, Yudai Fukui, Ko Ichimori, Hiroya Kuroyanagi

**Affiliations:** 1 Department of Gastroenterological Surgery, Toranomon Hospital, Tokyo, JPN; 2 Department of Gastroenterological Surgery, Saitama Red Cross Hospital, Saitama, JPN

**Keywords:** colorectal cancer, laparoscopic surgery, peritoneal dialysis, renal transplantation, case report

## Abstract

The development of transplantation technology has improved the prognosis of transplantation surgery; however, the negative impact of immunosuppressive drugs has increased the number of patients with cancer after transplantation. Recently, minimally invasive surgery has become more common for cancer treatment. We report our experience of performing laparoscopic sigmoid colon resection for a patient with a history of two renal transplantations and peritoneal dialysis. A 42-year-old male patient who developed purpura nephropathy underwent renal transplantation at ages eight and 34 years. He had been on peritoneal dialysis for five years before the second transplantation. The patient was referred to our department with the chief complaint of sudden abdominal pain. After an examination of imaging, we obtained a diagnosis of sigmoid colon cancer. Despite a history of peritoneal dialysis, laparoscopic sigmoid colon resection was successfully performed without complications after confirming that there were no adhesions in the abdominal cavity. The left lower port position had to be adjusted because the transplanted kidney protruded into the left iliac fossa. No postoperative complications and graft loss occurred. In this case, laparoscopic surgery was effective in lowering the risk of damage to the transplanted kidney and safely performing the procedure. The number of colorectal cancer cases in renal transplant patients is expected to increase, and some of these patients will have a history of peritoneal dialysis, which may make surgery more difficult. The successful outcome of this case highlights that laparoscopic surgery could be viable for patients with such a complex medical history.

## Introduction

The increased incidence of colorectal cancer after kidney transplantation has been widely reported in the literature [1,2]. Furthermore, the number of malignancies in patients who have undergone transplantation is expected to increase with the improvement in organ transplantation outcomes. Surgery for patients with a history of peritoneal dialysis and kidney transplantation can be complicated by adhesions and peritoneal membrane deterioration [3]. Nevertheless, surgical resection remains imperative for the curative management of resectable colorectal cancer. To the best of our knowledge, cases involving laparoscopic surgery for colorectal cancer after multiple renal transplantations and peritoneal dialysis have not been reported. We present an extremely rare case of a patient with sigmoid colon cancer who underwent resection after two renal transplantations and peritoneal dialysis.

## Case presentation

A 42-year-old Japanese man underwent a living donor kidney transplantation in the right iliac fossa because of purpura nephropathy at eight years old. Subsequently, he required peritoneal dialysis owing to graft failure at 29 years old. Transplantation with a deceased donor kidney was performed in the left iliac fossa at 34 years old, and the peritoneal dialysis catheter was removed at 35 years old. Immunosuppressive therapy comprised tacrolimus (1.5 mg/day), mizoribine (1 mg/day), and prednisolone (3 mg/day). The left graft function remained stable and was free of rejection. His medical history included total parathyroidectomy and autologous gland transplantation for secondary hyperparathyroidism at 30 years old. He had no history of allergies, smoking, or alcohol consumption.

**Figure 1:**
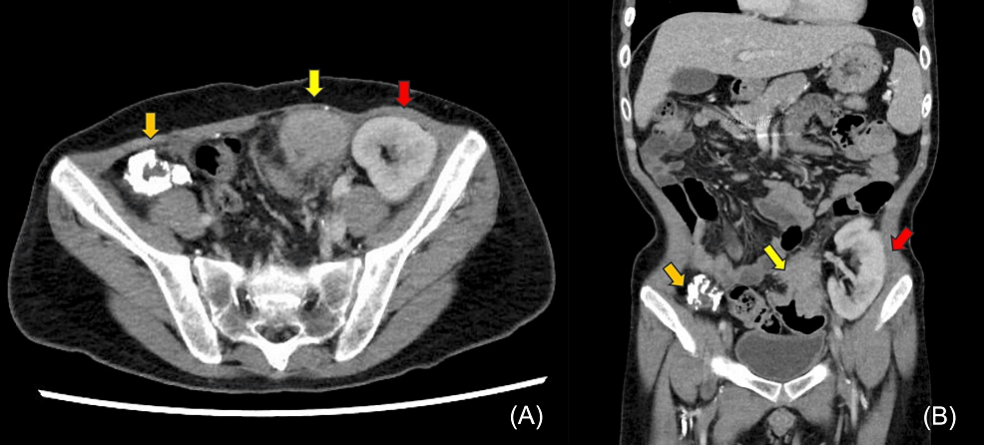
CT findings A CT scan revealed circumferential wall thickening with irregular surface and contrast enhancement on the sigmoid colon (yellow arrow) and near the left transplanted kidney (red arrow). The right transplanted kidney was atrophic and calcified (orange arrow). (A) Axial view. (B) Coronal view.

In 2022, eight years after the second transplantation, the patient presented to the emergency department of Toranomon Hospital, Japan, Tokyo, with sudden abdominal pain. On admission, his abdomen was flat and soft, and abdominal pain improved by using acetaminophen. CT showed circumferential wall thickening with an irregular surface and contrast enhancement in the sigmoid colon, and we suspected sigmoid colon cancer as the cause of the abdominal pain. The transplanted kidneys were observed in the bilateral pelvic cavities, and the right kidney transplant was atrophic and calcified (Figures 1A-1B). No evidence of lymph node or distant metastases was observed. 

**Figure 2:**
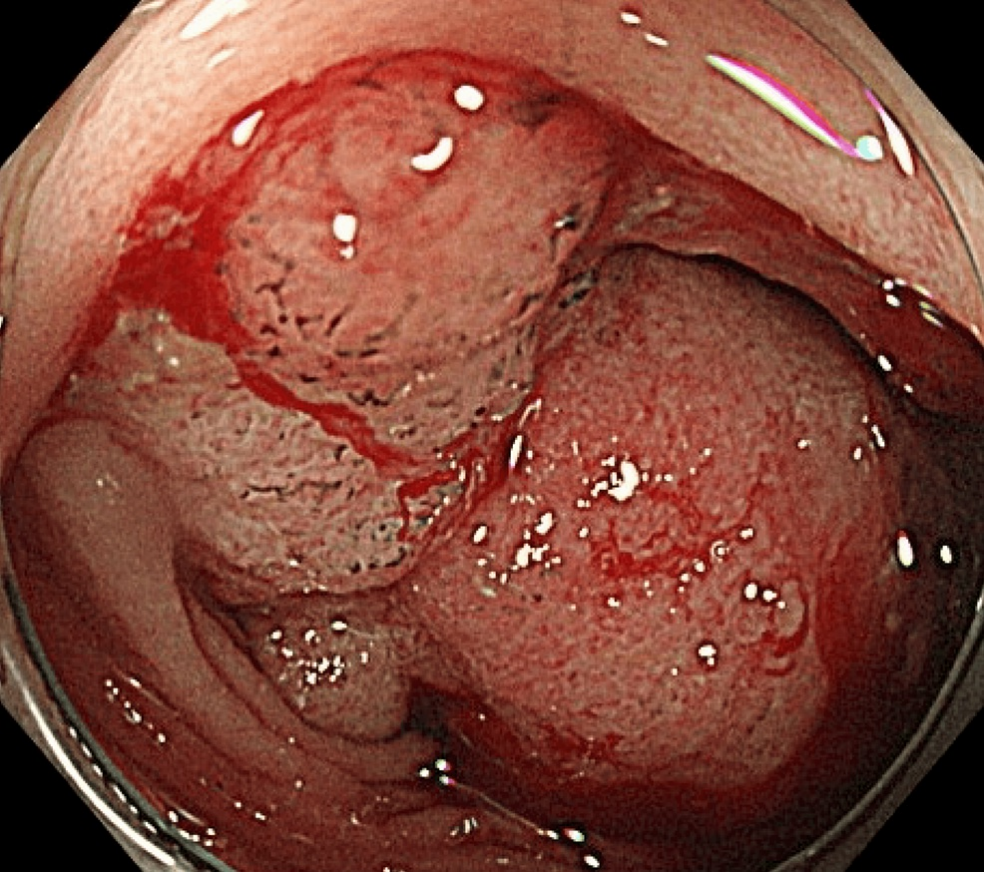
Colonoscopy finding A circumferential mass at the sigmoid colon obstructing the whole of the lumen.

A subsequent colonoscopy revealed a circumferential mass that obstructed the sigmoid colon, thus precluding observation beyond the tumor site (Figure 2). Biopsy results confirmed that the tumor was a tubular adenocarcinoma.

Laboratory data indicated mildly elevated inflammatory markers (WBC count of 7,300/μL; C-reactive protein level of 1.22 mg/dL) and no anemia. The patient’s serum creatinine level was 1.08 μmol/L, and serum tumor markers (carcinoembryonic antigen (CEA) and CA19-9) were within normal limits. 

Despite the proximity of the tumor to the left transplanted kidney, the retroperitoneal layer was preserved, suggesting noninvasiveness, and the tumor was considered resectable. Obstructive colitis was considered because of the presence of abdominal pain. Therefore, we performed semi-emergent surgery on the fifth day after admission. Because of the patient’s history of peritoneal dialysis and the possibility of severe intra-abdominal adhesions, the plan was to proceed promptly to laparotomy if laparoscopic surgery was difficult based on intraoperative findings. The patient took immunosuppressive drugs until the morning of surgery and resumed taking them from the first day after the operation.

**Figure 3:**
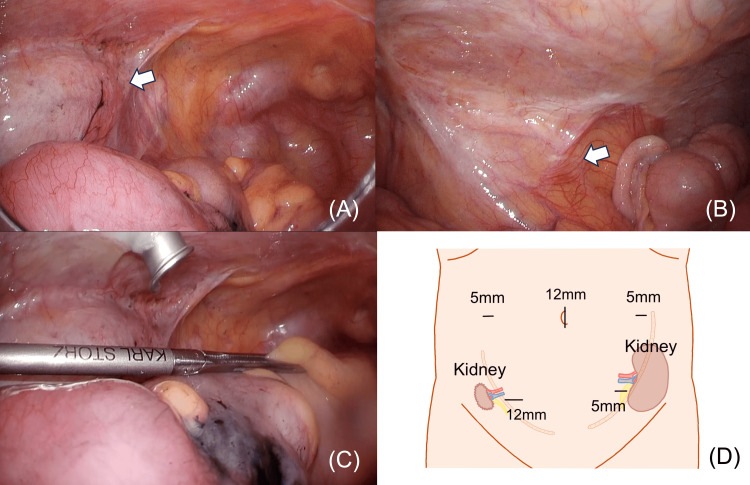
Intraoperative findings (A) The left transplanted kidney(arrow) was in the left lower abdomen. (B) The right transplanted kidney (arrow) graft was less prominent. (C) The left lower port was inserted more towards the midline than usual to avoid the transplanted kidney. (D) Schema depicts port placement and bilateral transplanted kidneys. This was created using Procreate®.

After general anesthesia was administered, the patient was placed in the lithotomy position. A 12-mm port was inserted through the transumbilical incision for placement of the operative laparoscope using the open method. The abdomen was insufflated with medical CO2 to 8 mmHg. Complete exploration of the abdominal cavity revealed no evidence of liver or peritoneal metastases. The tumor was located at the sigmoid colon, and the left transplanted kidney was identified in the left lower abdomen (Figure 3A). The right kidney graft was less prominent (Figure 3B). As there was no evidence of adhesions that were anticipated preoperatively, we decided to perform laparoscopic surgery. As usual, we inserted a 5-mm port in the right and left upper abdomen and a 12-mm port in the right lower abdomen. The right transplanted kidney did not affect port placement in the right lower abdomen. To safeguard the left transplanted kidney, the lower left port was positioned more medially than the standard port (Figure 3C,3D).

**Figure 4:**
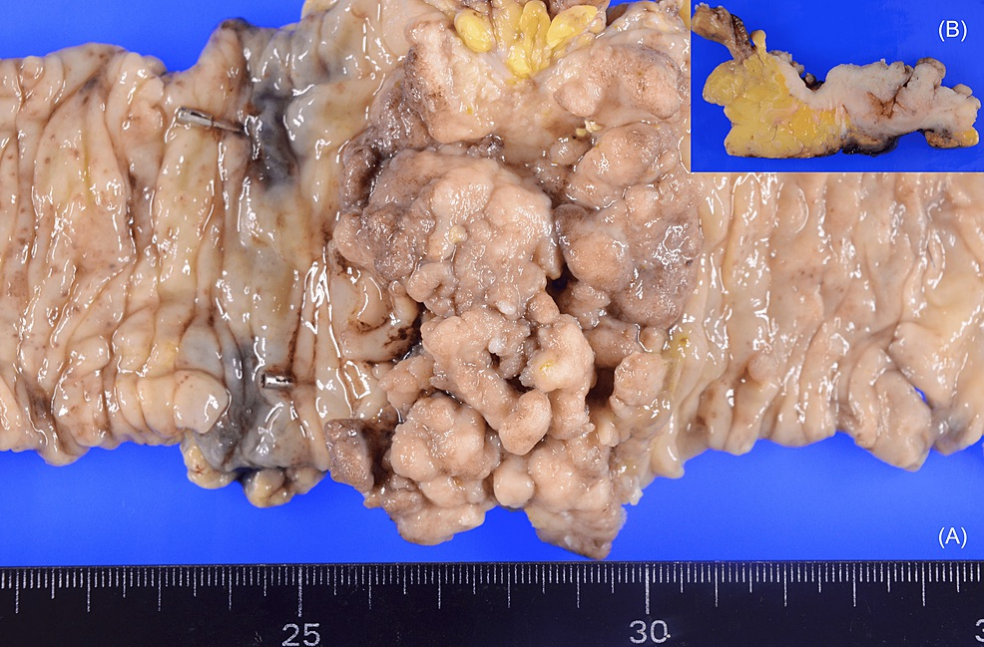
The specimen (A) The specimen was a type 2 circumferential tumor measuring 78 × 50 mm. (B) The tumor had grown through the muscularis propria and into the subserosa.

Normally, a 12-mm port would be used in the left lower abdomen. However, in this case, a 5-mm port was chosen because of the possibility of damage to the transplanted kidney and the port's mobility. The large tumor made it difficult to raise the sigmoid colon mesentery; however, the other surgical procedures were possible as usual. Complete tumor removal with tumor-free margins and tumor-specific mesorectal excision were performed. The inferior mesenteric artery was cut at the root, and the D3 lymph nodes were harvested. Surgery was completed successfully, without damage to the transplanted kidney, with an operative time of 228 minutes and blood loss of 50 mL. A circumferential tumor specimen with a measurement of 78 × 54 mm was obtained (Figure 4A, 4B).

**Figure 5:**
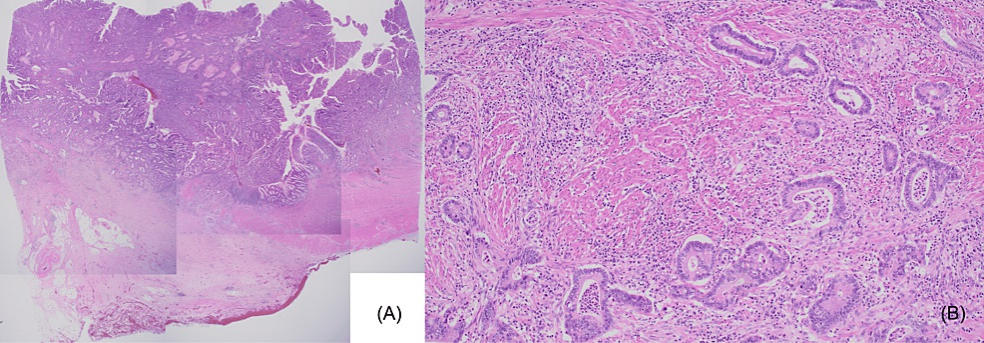
Histopathology images (hematoxylin and eosin stain) A histopathological examination revealed a moderately to well-differentiated adenocarcinoma with invasion of the subserosa. (A) At a 2x optical magnification. (B) At a 40x optical magnification.

A histopathological examination revealed a moderately to well-differentiated adenocarcinoma with invasion of the subserosa (Figure 5A, 5B). Tumor-free resection margins and the absence of lymph node metastases(0/31) indicated a tumor stage of pT3N0M0.

No postoperative complications occurred, and the patient was discharged from the hospital nine days after surgery. During the 20 months of follow-up, allograft rejection, complications, and recurrence were not observed.

## Discussion

There have been several reports of risk factors for malignancy after kidney transplantation. In addition to advanced age, white race, and history of malignancy, other factors such as an extensive duration after transplantation [1] and prolonged dialysis before transplantation [2] have been suggested as risk factors. Furthermore, the use of immunosuppressive drugs by recipients of kidney transplants has been implicated as a risk factor [4]. Kasiske et al. [5] reported that the risk of colorectal cancer was two times higher than that of the general population during the first year after transplantation and that it was 2.2 times higher during the third year after transplantation. More than 30 years have passed since our patient underwent the initial kidney transplantation. Therefore, long-term exposure to immunosuppressive therapy may have played a role in the development of the cancer.

Although there have been several reports of surgery for colorectal cancer after renal transplantation, only one case involving surgery after two renal transplantations has been reported [6]. A male patient underwent renal transplantation for end-stage renal disease caused by interstitial nephritis at 54 years old. At 63 years old, he underwent left renal transplantation because of graft failure. At 68 years old, upper rectal cancer was diagnosed, and he underwent open anterior resection. The postoperative course was uneventful. The patient eventually died of metastatic cancer 31 months after surgery; however, his organ grafts functioned well until the time of death.

Four cases involving laparoscopic surgery for colorectal cancer after kidney transplantation have been reported [7-10] by a case series comprising 19 patients with colorectal cancer after kidney transplantation who underwent laparoscopic or robotic surgery (Table 1).

**Table 1:** Laparoscopic surgery for colorectal cancer after kidney transplantation LAR: laparoscopic anterior resection; LHO: laparoscopic Hartmann’s operation; LLAR: laparoscopic low anterior resection; LLHC: laparoscopic left hemicolectomy; LRHC: laparoscopic right hemicolectomy; LSR: laparoscopic sigmoid resection; N/A: not available; RLAR: robotic low anterior resection

Author (reference number)	Age (years old)	Sex	Operative procedure	Operative time(min)	Blood loss(mL)	Postoperative hospital stays (days)	Complications
Alasari et al. [7] (n = 10)	56.8 (47-72)	Male: 5; female: 5	4LAR+3LLAR+1RLAR+2LRHC+1LLHC	192.5±53.87	30±53.74	9.7±5.81	Wound seroma chyloperitoneum: 1
Rivas et al. [8] (n = 3)	65 (54-73)	Male: 1; female: 2	2LSR+1LRHC	102.7±2.52	N/A	5.3±1.53	None
Xia et al. [9] (n = 5)	60.2 (51-76)	Male: 4; female: 1	4LLAR+1LSR	144±25.10	105±18.03	8.8±0.84	Anastomotic hemorrhage: 1; urinary tract infection: 1
Fujinaga et al. [10] (n = 1)	71	Male	1LHO	206	60	30	None
Our case (n = 1)	42	Male	1LSR	228	50	9	None

One kidney transplantation was performed for each of these four cases. The operative time and blood loss were not inferior to those of open surgery, and the hospital length of stay was significantly shorter for those who underwent laparoscopic surgery. Additionally, no major postoperative complications or loss of the transplanted kidneys occurred. The long-term prognosis was also favorable. Alasari et al. [7] reported that among the 10 patients who underwent laparoscopic or robotic surgery for colorectal cancer after kidney transplantation, the three-year disease-free rate was 83.3%, and the three-year overall survival rate was 100%.

Surgery for patients who have undergone kidney transplantation can be difficult because of the narrowing of the abdominal cavity caused by the transplanted kidney. Proceeding with surgery with a limited field of vision increases the risk of damage to the transplanted kidney. The magnifying effect of laparoscopic surgery is effective in selecting an appropriate dissection layer, even when the tumor is near the transplanted kidney. For our patient, preoperative imaging showed that the tumor was close to the left transplanted kidney. Combined partial nephrectomy was considered for radical resection in the case of invasion; however, no evidence of kidney invasion was observed intraoperatively. The magnifying effect of the laparoscope allowed for safe dissection and manipulation of the tumor and preservation of the left transplanted kidney. Moreover, during laparoscopic surgery, the transplanted kidney is shifted caudally by pneumoperitoneal pressure to provide adequate space for the working ports [8]. Lower ports proximal to the transplanted kidney can be easily placed under direct visualization [8]. In our case, the location of the transplanted kidney was confirmed under direct visualization, and insertion of the lower left port was achieved easily and safely. The presence of an atrophic right transplanted kidney minimally influenced the location of the right lower port.

Additionally, renal transplant patients are on long-term immunosuppressive medications, and laparotomy for these patients leads to delayed wound healing and increased risk of wound infection [11]. Laparoscopic surgery for renal transplant patients is also effective in this regard.

There have been several reports of laparoscopic surgery for patients on peritoneal dialysis with colorectal cancer, which has been successfully performed with good outcomes and early peritoneal dialysis resumption postoperatively [3]. However, there are few surgical reports on groups whose peritoneal dialysis was interrupted by successful renal transplantation or conversion to hemodialysis, and much remains unknown about the impact of peritoneal dialysis on the abdominal cavity.

The most serious complication associated with peritoneal dialysis is encapsulating peritoneal sclerosis (EPS), which is a clinical syndrome characterized by intestinal encapsulation with subsequent obstruction of the intestinal tract by the formation of excessive peritoneal fibrotic tissue [12]. According to a prospective study conducted in Japan, the incidence of EPS was 0%, 0.7%, 2.1%, 5.9%, 5.8%, and 17.2% in patients who had undergone peritoneal dialysis for three, five, eight, 10, 15, and more than 15 years, respectively [13]. It suggested that the frequency of EPS correlated with the duration of peritoneal dialysis. The main symptoms of EPS include decreased appetite, nausea/vomiting, diarrhea, and abdominal pain [14]. In advanced stages, both intestinal lumen narrowing and peritoneal calcification can be observed on CT [14].

Although much remains to be clarified regarding the pathophysiology of EPS secondary to peritoneal dialysis, it has been suggested that peritoneal decompensation because of peritoneal injury, such as a history of open surgery or intraperitoneal infection, is associated with the development of EPS [15]. The patient, in this case, had been on peritoneal dialysis for five years before his second successful kidney transplant. However, no physical findings suggested that EPS and CT showed no evidence of dilated bowel or peritoneal calcification. The patient did not have a history of open surgery, or an intra-abdominal infection may have caused EPS. Intraoperative evidence of severe adhesion was not observed; therefore, we could perform laparoscopic surgery.

We were able to complete laparoscopic surgery for a colorectal cancer patient who had undergone two renal transplants and a peritoneal dialysis. However, before performing surgery on a patient with such a complex history, we should consider all possibilities preoperatively. Our department performs over 500 laparoscopic colorectal resections per year. Our hospital also has four transplant surgeons on staff who perform approximately 50 kidney transplants annually, including five donated kidney transplants. However, we did not experience laparoscopic surgery for a patient with a complicated history, such as the present case. Therefore, we had a thorough preoperative discussion with nephrologists and transplant surgeons. We were prepared to promptly request support from the transplant surgeons if a high degree of adhesion was observed after the start of surgery or if it was determined that a complicated resection of a transplanted kidney was necessary. Further case accumulation is needed to determine if laparoscopic surgery can be performed in similar cases in the future. However, when laparoscopic surgery is feasible, it is a viable option to reduce the risk of damage to the transplanted kidney and lower the risk of complications such as delayed wound healing.

## Conclusions

We reported the first case of laparoscopic surgery for colorectal cancer in a patient with a history of two renal transplantations and peritoneal dialysis. Intraoperative findings showed no intra-abdominal adhesions, and pneumoperitoneal pressure provided sufficient working space. The port was safely inserted with direct observation of the transplanted kidney. The magnifying effect of the laparoscope allowed safe dissection of the area around the tumor, even near the transplanted kidney. While further study is required to examine the efficacy of laparoscopic surgery in this patient population, the successful outcome of this case highlights that laparoscopic surgery is a safe, viable, and practical choice, even for patients with a history of multiple renal transplantations and peritoneal dialysis.
